# Comparative Effectiveness of Bivalent (Original/Omicron BA.4/BA.5) COVID-19 Vaccines in Adults

**DOI:** 10.3390/vaccines11111711

**Published:** 2023-11-11

**Authors:** Hagit Kopel, Van Hung Nguyen, Catherine Boileau, Alina Bogdanov, Isabelle Winer, Thierry Ducruet, Ni Zeng, Mac Bonafede, Daina B. Esposito, David Martin, Andrew Rosen, Nicolas Van de Velde, Sten H. Vermund, Stefan Gravenstein, James A. Mansi

**Affiliations:** 1 Moderna, Inc., Cambridge, MA 02139, USAdaina.esposito@modernatx.com (D.B.E.); andrew.rosen@modernatx.com (A.R.);; 2 VHN Consulting Inc., Montreal, QC H2V 3L8, Canada; 3 Veradigm, Chicago, IL 60654, USA; 4 Yale School of Public Health, Yale University, New Haven, CT 06510, USA; sten.vermund@yale.edu; 5 Alpert Medical School and School of Public Health, Brown University, Providence, RI 02903, USA

**Keywords:** mRNA-1273.222, BNT162b2 Bivalent vaccine, bivalent vaccine, COVID-19 vaccine, hospitalization, symptomatic disease, relative vaccine effectiveness

## Abstract

The emergence of Omicron variants coincided with declining vaccine-induced protection against SARS-CoV-2. Two bivalent mRNA vaccines, mRNA-1273.222 (Moderna) and BNT162b2 Bivalent (Pfizer-BioNTech), were developed to provide greater protection against the predominate circulating variants by including mRNA that encodes both the ancestral (original) strain and BA.4/BA.5. We estimated their relative vaccine effectiveness (rVE) in preventing COVID-19-related outcomes in the US using a nationwide dataset linking primary care electronic health records and pharmacy/medical claims data. The study population (aged ≥18 years) received either vaccine between 31 August 2022 and 28 February 2023. We used propensity score weighting to adjust for baseline differences between groups. We estimated the rVE against COVID-19-related hospitalizations (primary outcome) and outpatient visits (secondary) for 1,034,538 mRNA-1273.222 and 1,670,666 BNT162b2 Bivalent vaccine recipients, with an adjusted rVE of 9.8% (95% confidence interval: 2.6–16.4%) and 5.1% (95% CI: 3.2–6.9%), respectively, for mRNA-1273.222 versus BNT162b2 Bivalent. The incremental relative effectiveness was greater among adults ≥ 65; the rVE against COVID-19-related hospitalizations and outpatient visits in these patients was 13.5% (95% CI: 5.5–20.8%) and 10.7% (8.2–13.1%), respectively. Overall, we found greater effectiveness of mRNA-1273.222 compared with the BNT162b2 Bivalent vaccine in preventing COVID-19-related hospitalizations and outpatient visits, with increased benefits in older adults.

## 1. Introduction

Despite high levels of vaccine effectiveness (VE) against the ancestral SARS-CoV-2 strain and variants observed in the early stages of the COVID-19 pandemic, the VE of a primary series of mRNA-1273 and BNT162b2 monovalent vaccines decreased with the emergence of the Delta and Omicron variants and subvariants [1,2]. Administration of a monovalent vaccine booster dose improved both immunogenicity and VE; however, effectiveness remained lower compared to that observed against earlier circulating SARS-CoV-2 variants.

To counter waning immunity and broaden protection against emerging variants, bivalent mRNA vaccines were developed targeting both the spike protein of the Omicron BA.4/BA.5 subvariant and the ancestral (original) SARS-CoV-2. These bivalent vaccines demonstrated increased protection against COVID-19-related symptomatic infections and severe outcomes and substantially boosted clinical protection and antibody titers against circulating variants for up to 6 months [3]. While the safety profile was similar between monovalent and bivalent mRNA vaccines, greater immunogenicity and relative vaccine effectiveness (rVE) were demonstrated with increased time since prior infection or prior COVID-19 vaccination [4,5,6,7,8,9,10,11,12,13]. Bivalent vaccines (Original/ Omicron BA.4/BA.5) were authorized in the United States (US) by the Food and Drug Administration (FDA) in August 2022 [14,15]. The bivalent vaccines were recommended by the US Centers for Disease Control and Prevention (CDC) for all adults in September 2022. Authorization for bivalent vaccines was subsequently extended to all individuals ≥6 months of age [16,17]. However, while approximately 70% of the eligible US population had completed a primary series, only ~17% of those eligible have received bivalent vaccination as of June 2023 [18].

While hospitalization rates during the period of Omicron predominance have been lower than those observed earlier in the pandemic [18], vulnerable sub-groups, including unvaccinated adults, older adults, immunocompromised persons, and those with certain chronic underlying medical conditions, remain at increased risk for severe outcomes and death. Hence, information on VE and waning protection over time has immediate public health implications [19] and is essential for COVID-19 vaccine decision-makers and healthcare providers to provide recommendations and increase vaccine confidence among patients, particularly those at higher risk for COVID-19-related morbidity and mortality [18,20,21].

Previous analyses of the primary series and booster reported slightly higher VE for the monovalent mRNA-1273 (Moderna) vaccine compared with BNT162b2 (Pfizer-BioNTech) in preventing both reported cases and COVID-19-related hospitalizations and outpatient visits [22,23,24,25]. In addition, a recent publication demonstrated a reduced risk of potential vaccine-related adverse events following mRNA-1273 vaccination compared with BNT162b2 in community-dwelling older adults [26]. As the rVE of bivalent mRNA vaccines has not been established, the current study evaluated data from a large nationwide dataset to estimate the rVE of the mRNA-1273.222 bivalent vaccine (Original/ Omicron BA.4/BA.5) and the BNT162b2 Bivalent vaccine (Original/ Omicron BA.4/BA.5) against COVID-19-related hospitalizations and outpatient visits in adults in the US.

## 2. Methods

### 2.1. Data Source and Deidentification

We performed an observational, retrospective cohort study using real-world data from two integrated sources: widely used primary care electronic health record (EHR) platforms in the US (the Veradigm EHR dataset, including the Allscripts Tier 1, Allscripts Tier 2, and Practice Fusion EHRs) and pharmacy and medical claims data (the Komodo dataset). Further details of the dataset and deidentification processes are provided in the supplementary materials. 

### 2.2. Participants and Study Design

This retrospective observational study was designed, implemented, and reported in accordance with Good Pharmacoepidemiological Practice, applicable local regulations, and the ethical principles laid down in the Declaration of Helsinki.

**Figure 1:**
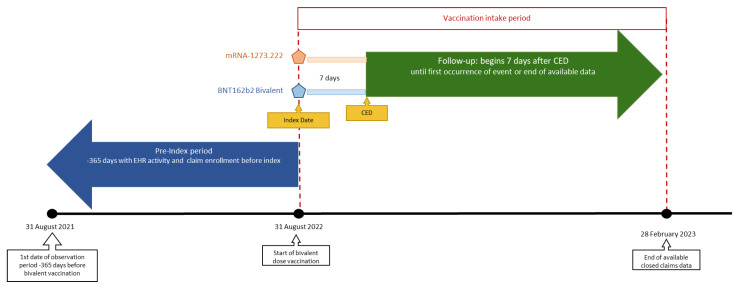
Study design. CED, cohort entry date; EHR, electronic health record.

Adults ≥18 years of age who had received either mRNA-1273.222 (50 mcg) or BNT162b2 Bivalent (30 mcg) between 31 August 2022 and 28 February 2023 were eligible for inclusion. We defined the index date as the vaccination date with the bivalent vaccine, and the cohort entry date (CED) as 7 days later (Figure 1). Individuals were followed up for outcomes of interest from the CED until the end of the available data (28 February 2023), receipt of another COVID-19 vaccine, or disenrollment from their medical/pharmacy plan, whichever occurred first. We classified individuals into two exposure cohorts based on the type of bivalent vaccine received (Supplementary Materials Table S1, list of identifying codes), with each individual only included once. 

Inclusion criteria included a minimum of 365 days of continuous medical and pharmacy enrollment in a health plan contributing data to the claim set prior to receipt of the booster (index date), and at least one contact with a health service provider in the 365 days prior to the index date. Exclusion criteria included evidence of SARS-CoV-2 infection or vaccination within the first 7 days after receipt of the bivalent vaccine, less than 1 day of follow-up, or missing birth year or sex.

### 2.3. Study Objectives

The primary objective of this study was to assess the rVE between mRNA-1273.222 and BNT162b2 Bivalent in preventing COVID-19-related illness requiring hospitalization, evaluated from the cohort entry date to the first occurrence. The secondary objective was to assess the rVE in preventing COVID-19-related outpatient visits, evaluated from cohort entry date to first occurrence (see Supplementary Materials Table S2). Exploratory objectives included subgroup analysis for primary and secondary outcomes (COVID-19-related hospitalizations and outpatient visits, respectively) by age group (≥50 years and ≥65 years).

### 2.4. Statistical Analysis

Using a multivariable logistic model for baseline variables, we calculated a propensity score and estimated weights to minimize the influence of potential confounders that we had identified prior to the analysis (Table S3). We then used stabilized and truncated weights to re-weight the study sample using a 1% asymmetrical trim to limit the effects of extreme weights on the study sample and performed weighting separately for the subgroup analysis by age group.

Using Cox regression models, we estimated unadjusted hazard ratios (HR) for the primary and secondary outcomes after propensity score weighting with exposure as the only predictor. Using multivariate Cox regression models, we then estimated the adjusted HRs, with any baseline variable with a standardized mean difference (SMD) of >0.1 after weighting included as a covariate. We calculated rVE as 100 × (1 − HR) for both unadjusted and adjusted estimates with 95% confidence intervals (95% CI). Further details of the statistical analysis are provided in the supplementary materials. All statistical analyses were performed using SAS 9.4 or R Statistical Software (v4.1.3) [27] survival package (v3.2-13).

### 2.5. Sensitivity Analyses

We performed two sensitivity analyses. First, since the main analyses were conducted on closed claims, we compared open versus closed claims (see Supplementary Materials for further details). Second, we used a cohort entry date of 14 days after vaccination (index date) instead of 7 days, as specified in the main closed claims analysis.

## 3. Results

**Figure 2:**
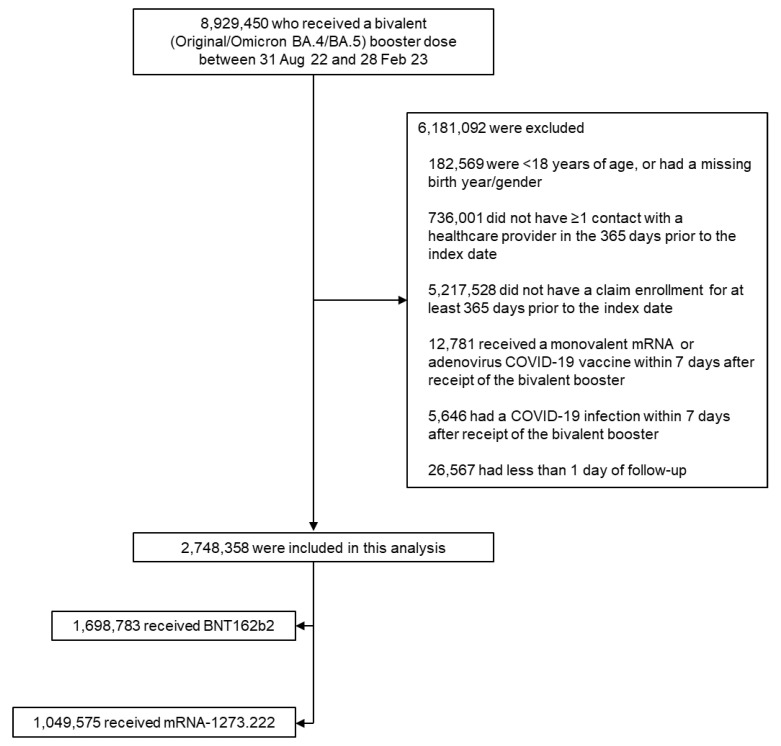
Selection of study populations: bivalent vaccine recipients for inclusion in the analysis. Note: exclusion criteria were evaluated in a stepwise fashion, summing to the total excluded.

Records were available for 8,929,450 individuals who had received a bivalent mRNA vaccine within the study timeframe (Figure 2). Of these, 2,748,358 individuals were eligible for inclusion in the pre-weight cohort, with 1,049,575 in the mRNA-1273.222 group and 1,698,783 in the BNT162b2 Bivalent group. After weighting, a total of 1,034,538 and 1,670,666 were included in the mRNA-1273.222 and BNT162b2 Bivalent groups, respectively (Table 1). After weighting, key baseline demographic variables were broadly similar between the two groups: the mean age was 58–59 years across groups, 58% were women, and 40% were white (where race was available). The majority (95%) of individuals in both groups had received the bivalent vaccine between September and December 2022 (Figure 1). The median duration of follow-up was 108 and 107 days for the mRNA-1273.222 and BNT162b2 Bivalent vaccines, respectively (Table 1). Overall, 69.5% of individuals vaccinated with mRNA-1273.222 and 68.2% of individuals vaccinated with BNT162b2 Bivalent had underlying medical conditions. As expected, we found a nearly identical hospitalization risk pattern in the evaluation of the negative control for both vaccines, with a *p*-value of 0.46, indicating that the groups were well-balanced (Figure 2). The baseline characteristics of the age subgroups are provided in Tables S4 and S5.

### 3.1. rVE against Hospitalized and Outpatient COVID-19

Overall, 1032 (0.10%) individuals who had received mRNA-1273.222 and 1855 (0.11%) who had received BNT162b2 Bivalent were hospitalized for COVID-19. Of these, 91.2% and 92.5% were ≥50 years of age, and 71.6% and 74.5% were ≥65 years of age at the index date for the two vaccines, respectively. Over three-quarters reported no previous documented COVID-19 infection (78.2% and 76.2% for the mRNA-1273.222 and BNT162b2 Bivalent vaccines, respectively), and 49.0% and 47.0%, respectively, had last received a monovalent COVID-19 vaccine >180 days previously. The most common underlying medical conditions with increased risk for severe COVID-19 outcomes in hospitalized patients were cancer (mRNA-1273.222: 42.3%; BNT162b2 Bivalent: 44.6%), heart conditions (46.6%; 49.0%), and diabetes (40.4%; 44.0%) (Table S6).

The rVE against COVID-19-related hospitalizations was 9.8% (95% CI: 2.6–16.4%; *p* = 0.008) for mRNA-1273.222 compared with BNT162b2 Bivalent across the entire study population. Differences between vaccines persisted over the 6 months post-vaccination (Figure S3). The estimate of the rVE against COVID-19-related outpatient visits was 5.1% (95% CI: 3.2–6.9%; *p* < 0.001) for mRNA-1273.222 compared with BNT162b2 Bivalent (Table 2). As cohorts were well-matched after inverse probability of treatment weighting, no covariates were included in the multivariable models for the overall population; therefore, estimates for both the unadjusted and adjusted rVE were equal.

### 3.2. rVE by Age Group

As the study met both the primary and secondary endpoints for the overall population, subgroup analysis was performed by age group. The rVE against both COVID-19-related hospitalizations and outpatient visits increased with increasing age, with adjusted rVEs of 11.0% (95% CI: 3.7–17.7%; *p* = 0.004) and 7.3% (5.3–9.3%; *p* < 0.0001) for hospitalizations and outpatient visits, respectively, in the ≥50 years subgroup, and 13.5% (95% CI: 5.5–20.8%; *p* = 0.001) and 10.7% (8.2–13.1%; *p* < 0.001) in the ≥65 years subgroup, respectively (Table 3).

### 3.3. Sensitivity Analyses

Point estimates of rVEs from evaluation of open claims were similar to those seen in the closed claims analysis (n = 3,065,980 for mRNA-1273.222 and n = 4,703,189 for BNT162b2 Bivalent): 12.9% (95% CI: 9.1–16.4%) and 5.8% (4.7–6.9%) against COVID-19-related hospitalizations and outpatient visits, respectively (Table 3).

Extending the time between vaccination (index date) and cohort entry date from 7 to 14 days in the closed claims analysis resulted in rVE estimates of 10.6% (95% CI: 3.1–17.5%) and 4.6% (2.6–6.5%) against COVID-19-related hospitalizations and outpatient visits, respectively (Table 3).

## 4. Discussion

This is the first study that directly evaluates the rVE of bivalent mRNA vaccines containing the ancestral (original) and omicron (BA.4/BA.5) strains in the prevention of COVID-19-related hospitalizations and outpatient visits. In this retrospective analysis of real-world data on more than 2.5 million vaccinated individuals, the mRNA-1273.222 bivalent vaccine (Original/ Omicron BA.4/BA.5) appears significantly more effective than the BNT162b2 Bivalent vaccine (Original/ Omicron BA.4/BA.5) in preventing COVID-19-related hospitalizations and outpatient visits in adults. The rVEs against both outcomes increased with increasing age. This is particularly important given the disproportionate burden of COVID-19-related morbidity and mortality in older adults and expands on the recent findings of the reduced risk of potential vaccine-related adverse events following mRNA-1273 monovalent vaccination compared with BNT162b2 in community-dwelling older adults in the US [26].

The results of this study echo those observed following analysis of the primary series and monovalent booster in the same dataset [25]. Similar to the current study, incremental benefits in rVE against hospitalizations were observed following the monovalent booster with increasing age, with rVEs of 40% (95% CI: 11–60%) in adults ≥65 years and 12% (0–30%) in the 18 to 64 age group. Other studies have also shown the increased effectiveness of mRNA-1273 versus BNT162b2 boosters against symptomatic infections and severe outcomes [22,24]. In a matched cohort study comparing the effectiveness of a single monovalent booster dose of mRNA-1273 and BNT162b2 using the OpenSAFELY-TPP research platform in the UK, the hazard ratio of the risk of hospital admission at 20 weeks post-booster was 0.89 (95% CI: 0.82 to 0.95), indicating a modest benefit of mRNA-1273 [22]. In a similar matched cohort study of US veterans, risks of 16-week COVID-19 outcomes were also lower for mRNA-1273 compared with the BNT162b2 monovalent booster, with an excess number of COVID-19 hospitalization events over the 16-week post-booster period of 10.6 (95% CI: 5.1–19.7) for BNT162b2 compared with mRNA-1273 [24]. Moreover, while there was no formal comparison between the vaccines, a study evaluating the effectiveness of bivalent vaccines containing any omicron subvariant also showed higher effectiveness for mRNA-1273.222 versus BNT162b2 Bivalent against infection [28].

The observed differences in effectiveness between the mRNA bivalent vaccines, particularly in older adults, may be explained at least in part by differences in vaccine humoral and T-cell immunity. In terms of humoral immunity, mRNA-1273 induces a significantly greater antibody immune response compared with BNT162b2 [29], which is consistent against different variants of concern, persisting even 6–8 months following vaccination [30,31,32,33]. Moreover, this greater and more durable immune response has been demonstrated among older adults, with studies showing that age is negatively correlated with antibody levels in participants who were vaccinated with BNT162b2, but not among mRNA-1273 vaccinees. Vaccination with mRNA-1273 induced higher antibody levels with a corresponding lower waning rate that was not age-dependent, as opposed to the BNT162b2 cohort, in which older individuals generated lower antibody levels with a higher waning ratio when compared to young adults [31,34].

Antibodies that can rapidly opsonize pathogens and drive pathogen clearance via opsinophagocytosis or killing are key to protection against many other respiratory pathogens [35]. Thus, in addition to neutralizing antibodies, antibodies that bind to viral proteins may also contribute to the immune control of infection through the increased clearance of free viruses or by targeting infected cells for immune clearance [36]. Indeed, there is emerging evidence describing functional antibody differences between the vaccines, beyond neutralization, that may account for differential mucosal protection. mRNA-1273 has been shown to induce more opsonophagocytic and cytotoxic antibodies compared to BNT162b2, which may be important in the rapid capture and clearance of the virus from the mucosal tract [37]. Furthermore, following mRNA-1273 vaccination, higher levels of IgA were measured systemically compared to BNT162b2, which may be important for mucosal protection, aiding the immune response even when new viral variants may emerge that subvert the neutralizing antibody response [38]. Along these lines, systemic vaccine-induced IgA levels have been identified as a strong correlate of protection against viral breakthrough following boosting in the setting of high neutralizing antibody responses [39].

In addition to the humoral response, several studies have shown that mRNA-1273 elicits more robust and long-term T-cells response compared with BNT162b2 [40,41,42,43], with significantly greater memory CD4+ T-cell frequencies following immunization with mRNA-1273 compared with BNT162b2 [41]. The enhanced functional humoral immune response and T-cell immunity observed following vaccination with mRNA-1273 may contribute to the differences in effectiveness observed, especially in the older age groups who often have lower responses to immunization, in part related to immunosenescence [44,45].

In this study, we used data from two integrated sources. Utilization of a comprehensive real-world dataset that integrates various sources of patient information enables the assessment of outcomes that are not always examined in clinical trials and offers a broader perspective on effectiveness in a real-world setting. Combining integrated databases that link both EHR and claims data can provide a well-rounded perspective on an individual’s health status and utilization of health care services [46], together with allowing robust adjustments of the data for well-established confounders. In this study, the information on exposure, outcome, and covariates was retrospectively collected from patient records in a consistent manner across all exposure cohorts using specific codes, which reduces the likelihood of differential misclassification. and the conclusions from the main analysis were confirmed by planned sensitivity analyses.

As with all observational or quasi-experimental studies, this study also has limitations. First, we did not evaluate long-term vaccine effectiveness, as we had a follow-up period of up to 6 months post-booster available. Given the differences in antibody waning and durability observed in immunogenicity studies of earlier vaccine formulations [7,30,47], it might be that the rVE becomes more pronounced over a longer follow-up period. Future longer-term analysis could provide a broader overview of long-term comparative vaccine performance and help guide decision-making on the optimal interval. Additionally, the current primary analysis was restricted to closed claims, which provide a comprehensive overview of a patient’s healthcare interactions but may not capture all cases and can be limited in terms of the sample size. However, sensitivity analysis performed on the open claims database supported the findings from the closed claims database. Furthermore, in this study, we included individuals who had consulted healthcare providers during the previous 365 days, which excluded healthy individuals who received a vaccine but did not use healthcare services during the year prior to receipt, potentially biasing towards a more at-risk population. Older adults and those with underlying chronic health conditions, who have an increased risk of more severe COVID-19 outcomes, are more likely than average healthy adults to utilize healthcare services [48]. Thus, while our results may not be representative of a population of healthy individuals, they include the most vulnerable population. Finally, the results of the study should be interpreted within the context of its retrospective nature. As with the previous analysis on the primary series and monovalent boosters [25], there may have been differences between the two vaccine groups that were not fully accounted for by the pre-defined covariates and thereby confounded the rVE estimates. It is possible that individuals with greater risk for COVID-19-related severe outcomes were vaccinated with BNT162b2 Bivalent compared to mRNA-1273.222 due to its earlier availability. While this could introduce some residual bias, we do not believe that it is a significant concern. Our negative control test results demonstrated no difference between the groups in terms of hospitalizations, suggesting that patient behavior bias may not have influenced our findings. Additionally, the propensity scoring analysis ensured the groups were well-balanced in terms of known comorbidities, reducing the likelihood of differences in patient behavior based on risk factors.

In summary, both bivalent vaccines have been shown to provide substantial protection against COVID-19-related hospitalizations and outpatient visits. In this analysis, the mRNA-1273.222 vaccine (Original/ Omicron BA.4/BA.5) provided greater protection against hospitalizations and outpatient visits compared with BNT162b2 Bivalent (Original/ Omicron BA.4/BA.5) during a period of omicron BA.4/BA.5 predominance. On 11 September 2023, the US FDA authorized the updated XBB1.5 containing mRNA vaccines, and on 12 September 2023, the US ACIP recommended these vaccines for all individuals ages 6 months and older. These updated vaccines are consistent in terms of dosage (50 µg for Moderna and 30 µg for Pfizer) with the bivalent formulations evaluated in this study. Moreover, the vaccine compositions differ only in the mRNA sequence between the bivalent and updated vaccines for 2023 [49,50]. The results of this study can help guide decision-makers and raise the need to evaluate vaccine differences of current and future formulations to ensure optimized protection against continually emerging SARS-CoV-2 variants and subvariants.

## Figures and Tables

**Table 1:** Key baseline characteristics of patients included in the mRNA-1273.222 and BNT162b2 Bivalent vaccine groups (pre- and post-weighting).

		Pre-Weighting			Post-Weighting		
		mRNA-1273.222	BNT162b2 Bivalent	SMD	mRNA-1273.222	BNT162b2 Bivalent	SMD
Number of patients		1,049,575	1,698,783		1,034,538	1,670,666	
Age at index, mean (SD)		60 (16.3)	58 (16.9)	0.1091	59 (16.5)	58 (16.7)	0.0238
Sex	Female	604,099 (57.6)	989,261 (58.2)	0.0137	598,134 (57.8)	969,767 (58.0)	0.0047
	Male	445,476 (42.4)	709,522 (41.8)		436,404 (42.2)	700,899 (42.0)	
Race	Black	41,438 (3.9)	71,545 (4.2)	0.0242	41,695 (4.0)	68,926 (4.1)	0.0064
	Other	43,292 (4.1)	72,004 (4.2)		43,444 (4.2)	70,448 (4.2)	
	White	425,675 (40.6)	670,704 (39.5)		413,323 (40.0)	663,768 (39.7)	
	Unknown	539,170 (51.4)	884,530 (52.1)		536,076 (51.8)	867,524 (51.9)	
Ethnicity	Hispanic	38,672 (3.7)	66,301 (3.9)	0.0140	39,044 (3.8)	64,185 (3.8)	0.0046
	Non-Hispanic	852,545 (81.2)	1,371,939 (80.8)		838,544 (81.1)	1,351,443 (80.9)	
	Unknown	158,358 (15.1)	260,543 (15.3)		156,950 (15.2)	255,038 (15.3)	
Region	Midwest	201,029 (19.2)	373,067 (22.0)	0.0760	210,897 (20.4)	354,455 (21.2)	0.021
	Northeast	272,159 (25.9)	437,311 (25.7)		268,924 (26.0)	432,274 (25.9)	
	South	314,939 (30.0)	470,675 (27.7)		296,959 (28.7)	471,340 (28.2)	
	West	196,278 (18.7)	315,809 (18.6)		194,309 (18.8)	311,409 (18.6)	
	Unknown	65,170 (6.2)	101,921 (6.0)		63,449 (6.1)	101,188 (6.1)	
Month of index	August 2022	12 (<0.1)	21 (<0.1)	0.0645	13 (<0.1)	21 (<0.1)	0.0206
	September 2022	252,601 (24.1)	453,304 (26.7)		259,247 (25.1)	433,404 (25.9)	
	October 2022	385,320 (36.7)	619,160 (36.4)		380,441 (36.8)	609,256 (36.5)	
	November 2022	230,341 (21.9)	351,626 (20.7)		221,444 (21.4)	351,191 (21.0)	
	December 2022	128,551 (12.2)	194,995 (11.5)		123,180 (11.9)	196,156 (11.7)	
	January 2023	42,133 (4)	63,853 (3.8)		40,173 (3.9)	64,422 (3.9)	
	February 2023	10,617 (1)	15,824 (0.9)		10,041 (1.0)	16,215 (1.0)	
Primary series COVID-19 vaccine	Heterologous	90,742 (8.6)	179,627 (10.6)	0.1652	102,076 (9.9)	169,119 (10.1)	0.0297
	Homologous	301,861 (28.8)	369,852 (21.8)		252,916 (24.4)	387,485 (23.2)	
	Not reported	656,972 (62.6)	1,149,304 (67.7)		679,546 (65.7)	1,114,062 (66.7)	
Time since last COVID-19 monovalent vaccination	≤90 days	15,489 (1.5)	18,482 (1.1)	0.2365	12,849 (1.2)	20,336 (1.2)	0.0440
	91–180 days	215,483 (20.5)	216,131 (12.7)		163,208 (15.8)	238,465 (14.3)	
	>180 days	614,126 (58.5)	1,029,498 (60.6)		622,635 (60.2)	1,015,967 (60.8)	
	Not reported	204,477 (19.5)	434,672 (25.6)		235,845 (22.8)	395,898 (23.7)	
Time since last COVID-19 infection	≤120 days	34,542 (3.3)	56,609 (3.3)	0.0219	34,348 (3.3)	55,608 (3.3)	0.0059
	121–180 days	19,610 (1.9)	31,653 (1.9)		19,479 (1.9)	31,327 (1.9)	
	>180 days	72,675 (6.9)	127,046 (7.5)		74,147 (7.2)	122,306 (7.3)	
	Not reported	922,748 (87.9)	1,483,475 (87.3)		906,564 (87.6)	1,461,425 (87.5)	
Follow-up duration, days, median (IQR)		106 (70–134)	108 (71–137)		108 (70–135)	107 (71–137)	
Underlying medical conditions	Asthma	89,784 (8.6)	146,786 (8.6)	0.0031	88,608 (8.6)	143,799 (8.6)	0.0015
	Cancer	331,893 (31.6)	507,975 (29.9)	0.0373	317,902 (30.7)	506,655 (30.3)	0.0087
	Cerebrovascular disease	57,932 (5.5)	89,250 (5.3)	0.0118	55,150 (5.3)	88,816 (5.3)	0.0007
	Chronic lung disease	82,755 (7.9)	126,674 (7.5)	0.0161	78,570 (7.6)	126,028 (7.5)	0.0019
	Chronic liver disease	11,770 (1.1)	18,988 (1.1)	0.0003	11,499 (1.1)	18,673 (1.1)	0.0006
	CKD	90,134 (8.6)	138,166 (8.1)	0.0164	85,505 (8.3)	137,539 (8.2)	0.0012
	Cystic fibrosis	235 (<0.1)	343 (<0.1)	0.0015	217 (<0.1)	342 (<0.1)	0.0004
	Diabetes type 1 or 2	210,600 (20.1)	325,656 (19.2)	0.0225	201,683 (19.5)	323,800 (19.4)	0.0029
	Disability	69,181 (6.6)	117,573 (6.9)	0.0131	69,582 (6.7)	114,107 (6.8)	0.0041
	Heart conditions	145,354 (13.8)	220,523 (13.0)	0.0255	137,513 (13.3)	220,348 (13.2)	0.0030
	HIV	5471 (0.5)	8770 (0.5)	0.0007	5397 (0.5)	8639 (0.5)	0.0006
	Mental health disorders	166,566 (15.9)	284,385 (16.7)	0.0236	167,713 (16.2)	275,635 (16.5)	0.0078
	Neurological conditions	21,962 (2.1)	39,359 (2.3)	0.0153	22,279 (2.2)	37,628 (2.3)	0.0067
	Obesity	210,605 (20.1)	339,268 (20.0)	0.0024	206,336 (19.9)	333,651 (20.0)	0.0007
	Primary immunodeficiencies	69,102 (6.6)	107,213 (6.3)	0.0111	66,569 (6.4)	106,563 (6.4)	0.0023
	Pregnancy ^a^	2861 (0.3)	5465 (0.3)	0.0090	3109 (0.3)	5160 (0.3)	0.0015
	Physical inactivity	975 (0.1)	1608 (0.1)	0.0006	962 (<0.1)	1566 (<0.1)	0.0003
	Smoking ^b^	125,869 (12)	202,673 (11.9)	0.0019	122,902 (11.9)	199,274 (11.9)	0.0015
	Solid organ or hematopoietic stem cell transplant	9038 (0.9)	14,493 (0.9)	0.0009	8727 (0.8)	14,231 (0.9)	0.0009
	Tuberculosis	325 (<0.1)	537 (<0.1)	0.0004	323 (<0.1)	523 (<0.1)	0.0001
	Use of immunosuppressants	54,552 (5.2)	84,577 (5.0)	0.0100	52,537 (5.1)	84,130 (5.0)	0.0019

Data are presented as n (%) unless otherwise stated. CKD, chronic kidney disease; IQR, interquartile range; SD, standard deviation; SMD, standardized mean difference ^a^ Includes recent pregnancy. ^b^ Includes current and former smokers.

**Table 2:** Unadjusted and adjusted rVEs for mRNA-1273.222 versus BNT162b2 Bivalent for the overall study population.

COVID-19-Related Outcome	Unadjusted rVE	Adjusted rVE	*p* Value ^a^
Hospitalization	9.8% (2.6–16.4%)	9.8% (2.6–16.4%)	0.008
Outpatient	5.1% (3.2–6.9%)	5.1% (3.2–6.9%)	<0.0001

rVE, relative vaccine effectiveness. ^a^ Associated *p* values are given for the adjusted rVE, with secondary outcomes adjusted for multiple testing.

**Table 3:** Unadjusted and adjusted rVEs for mRNA-1273.222 versus BNT162b2 Bivalent for the subgroups and sensitivity outcomes.

COVID-19-Related Outcome	Unadjusted rVE	Adjusted rVE	*p* Value ^a^
**Subgroup analysis by age**			
**≥50 years**			
Hospitalization	11.0% (3.7–17.7%)	11.0% (3.7–17.7%)	0.004
Outpatient	7.3% (5.3–9.3%)	7.3% (5.3–9.3%)	<0.0001
**≥65 years**			
Hospitalization	13.5% (5.5–20.8%)	13.5% (5.5–20.8%)	0.001
Outpatient	10.7% (8.2–13.1%)	10.7% (8.2–13.1%)	<0.0001
**Sensitivity analysis**			
Open claims			
Hospitalization	12.9% (9.1–16.4%)	12.9% (9.1–16.4%)	<0.0001
Outpatient	5.8% (4.7–6.9%)	5.8% (4.7–6.9%)	<0.0001
**Cohort entry date 14 days post-vaccination closed claims**			
Hospitalization	10.6% (3.1–17.5%)	10.6% (3.1–17.5%)	0.006
Outpatient	4.6% (2.6–6.5%)	4.6% (2.6–6.5%)	<0.0001

rVE, relative vaccine effectiveness ^a^ Associated *p* values are given for the adjusted rVE, with secondary outcomes adjusted for multiple testing.

## Data Availability

Individual-level data reported in this study are not publicly shared. Upon request, and subject to review, Veradigm may provide the deidentified aggregate-level data that support the findings of this study. Deidentified data (including participant data as applicable) may be shared upon approval of an analysis proposal and a signed data access agreement. Individual-level data reported in this study are shared fully with regulatory agencies.

## Supplementary Materials

The following supporting information can be downloaded at: https://www.mdpi.com/article/10.3390/vaccines11111711/s1, Table S1. List of CVX, CPT, and NDC codes used to identify bivalent (Original/ Omicron BA.4/BA.5) COVID-19 vaccines from the Veradigm EHR and linked claims datasets. Table S2. ICD-10 and SNOMED codes for the outcome of COVID-19-related medical encounters identified from individuals’ primary care EHRs, pharmacy, and medical claims. Table S3. List of potential confounding baseline variables which were weighted using propensity weighting scoring prior to data analysis. Table S4. Baseline characteristics (post-weighting) of individuals ≥50 years vaccinated with the mRNA-1273.222 or BNT162b2 Bivalent vaccine. Table S5. Baseline characteristics (post-weighting) of individuals ≥65 years vaccinated with the mRNA-1273.222 or BNT162b2 Bivalent vaccine. Table S6. Baseline characteristics (post-weighting) of individuals hospitalized for COVID-19 vaccinated with the mRNA-1273.222 or BNT162b2 Bivalent vaccine. Figure S1. Percentage of individuals included in the weighted analysis by month of vaccination. Figure S2. Negative control Kaplan–Meier curves for COVID-19–related hospitalization rates 7 days prior to CED in recipients of the mRNA-1273.222 or BNT162b2 Bivalent vaccine. Figure S3. Kaplan–Meier curves of COVID-19–related hospitalizations over time in recipients of the mRNA-1273.222 or BNT162b2 Bivalent vaccine.
