# Supplementary material for: Comparative Effectiveness of Bivalent (Original/Omicron BA.4/BA.5) COVID-19 Vaccines in Adults
Source: Vaccines (Basel). 2023 Nov 11;11(11):1711. doi: 10.3390/vaccines11111711 (PMC10675676; doi:10.3390/vaccines11111711)
Supplement: Supplementary file 1 [file vaccines-11-01711-s001.zip › vaccines-2671427-supplementary.pdf]

Supplementary Materials

Supplementary Methods

Data set and deidentification

Past research successfully used this integrated dataset to evaluate vaccine effectiveness and its data to contain key variables for conducting effectiveness /outcomes research and demonstrated it to be representative of the US population [1–3]. Each data asset meets the minimum protected health information (PHI) data requirements as specified for use by the algorithm. Research staff were not involved in the preparation of datasets containing PHI or the actual running of the algorithm. The final linked data set merged the patient-level deidentified tokens in each individual dataset and contained no PHI. This linked, deidentified dataset was compliant with the Health Insurance Portability and Accountability Act (HIPAA) and certified for research use. Approval by an institutional review board and patient consent were not necessary as the study was a noninterventional, retrospective cohort using a certified HIPAA-compliant deidentified research database.

Statistical analysis

We considered the cohort to be well-balanced if the difference in SMD between the comparison groups was <0.1.

To explore the possibility of residual confounding, we used a negative control. We evaluated the risk of COVID-19-related hospitalization within the first 7 days after vaccination and compared the cumulative incidence among the exposure groups during this immunization lag period. This approach allowed us to ensure that both groups were comparable before the cohort entry date (CED).

To control inflation of the type 1 error rate due to multiple testing (family wise error rate), we used a step-down testing procedure for secondary endpoints and the age subgroup analysis by adjusting the rejection criteria for each of the individually tested hypotheses [4]. We did statistical testing at a significance level of p=0.05 and stopped when a secondary endpoint did not reach statistical significance.

Open and closed claims datasets

In this study, we have used closed claims in the main analysis and open claims in the sensitivity analysis. A three-tiered definition of closed claims was used to generate a closed claims dataset. First, a claim was closed for a patient if all medical and pharmacy transactions were received in a given time period. If not all transactions for a patient had been received, the data was considered "open" for that patient. Second, a source was "closed/payer complete" if all patient claims were "closed" under that payer. Third, time periods in which patients were closed could be determined by the enrollment dates; hence enrollment periods were considered closed if the end date of enrollment was closed for that patient. Medical and pharmacy claims included in a closed claims dataset were all post-adjudication claims. Therefore, the closed claims dataset that was used in the main analysis captured all claims associated with a patient (medical and pharmacy) during the time period in which they were enrolled. The open claims analysis used all claims available for a given patient, regardless of whether they were during an enrollment period, sourced from a payer, or considered closed. For

patients with open claims, follow-up was concluded at the end of data availability. The analysis utilized the last evidence of database activity (EHR or claims) as the endpoint for follow-up.

Identification of COVID-19-related medical encounters

We identified inpatient admissions based on all claims billed in the inpatient setting. Among these claims, we assigned those associated with inpatient hospital services to the inpatient hospital designation, and those associated with non-inpatient services (such as skilled nursing facilities or emergency department visits) to the outpatient designation. Once encounters were identified as either inpatient or outpatient, we flagged claims with a diagnosis code for COVID-19 (in any position). We identified additional outpatient cases in the EHR via diagnosis codes (see **Table S2**) and included telehealth consultations and emergency visits.

Table S1. **List of CVX, CPT, and NDC codes used to identify bivalent (Original/ Omicron BA.4/BA.5) COVID-19 vaccines from the Veradigm EHR and linked claims datasets.**

Code Type	Code	Description	Vaccine	Dose Sequence
CPT	91313	Severe acute respiratory syndrome coronavirus 2 (SARS-CoV-2) (coronavirus disease [COVID-19]) vaccine, mRNA-LNP, spike protein, bivalent, preservative free, 50 mcg/0.5 mL dosage, for intramuscular use	mRNA-1273.222	Bivalent booster
CPT	0134A	Immunization administration by intramuscular injection of severe acute respiratory syndrome coronavirus 2 (SARS-CoV-2) (coronavirus disease [COVID-19]) vaccine, mRNA-LNP, spike protein, bivalent, preservative free, 50 mcg/0.5 mL dosage, booster dose	mRNA-1273.222	Bivalent booster
NDC	80777-0280-05	mRNA-1273 COVID-19 vaccine, BL (80777-280-05)	mRNA-1273.222	Bivalent booster
NDC	80777-0280-99	mRNA-1273 COVID-19 vaccine, Bivalent; 0.1 mg/mL; 10 VIAL in 1 CARTON, 2.5 mL in 1 VIAL	mRNA-1273.222	Bivalent booster
CVX	229	SARS-COV-2 (COVID-19) vaccine, mRNA, spike protein, LNP, bivalent, preservative free, 50 mcg/0.5 mL dose [Pre-EUA mRNA-1273 bivalent adult booster original strain + omicron strain]	mRNA-1273.222	Bivalent booster
CVX	300	SARS-COV-2 (COVID-19) vaccine, mRNA, spike protein, LNP, bivalent booster, preservative free, 30 mcg/0.3 mL dose, tris-sucrose formulation; EUA authorized BNT162b2 adult bivalent booster ages 12+ yrs, original strain + omicron BA.4/BA.5. Not authorized by WHO. Non-US tradename for same formulation (Comirnaty Bivalent) counted toward immunity in US	BNT162b2 Bivalent	Bivalent booster
CPT	91312	Severe acute respiratory syndrome coronavirus 2 (SARS-CoV-2) (coronavirus disease [COVID-19]) vaccine, mRNA-LNP, bivalent spike protein, preservative free, 30 mcg/0.3 mL dosage, tris-sucrose formulation, for intramuscular use	BNT162b2 Bivalent	Bivalent booster

Code Type	Code	Description	Vaccine	Dose Sequence
CPT	0124A	Immunization administration by intramuscular injection of severe acute respiratory syndrome coronavirus 2 (SARS-CoV-2) (coronavirus disease [COVID-19]) vaccine, mRNA-LNP, bivalent spike protein, preservative free, 30 mcg/0.3 mL dosage, tris-sucrose formulation, booster dose	BNT162b2 Bivalent	Bivalent booster
NDC	59267-0304-01	PFIZER COVID BIVALENT BOOST(12Y UP)(ORIG-BA.4/5)(GRAY)(EUA)	BNT162b2 Bivalent	Bivalent booster
NDC	59267-0304-02	PFIZER COVID BIVALENT BOOST(12Y UP)(ORIG-BA.4/5)(GRAY)(EUA)	BNT162b2 Bivalent	Bivalent booster
NDC	59267-1404-01	PFIZER COVID BIVALENT BOOST(12Y UP)(ORIG-BA.4/5)(GRAY)(EUA)	BNT162b2 Bivalent	Bivalent booster
NDC	59267-1404-02	PFIZER COVID BIVALENT BOOST(12Y UP)(ORIG-BA.4/5)(GRAY)(EUA)	BNT162b2 Bivalent	Bivalent booster

CPT, current procedural terminology; NDC, national drug codes; CVX, vaccine administered code set.

Table S2. ICD-10 and SNOMED codes for the outcome of COVID-19-related medical encounters identified from individuals' primary care EHRs, pharmacy, and medical claims.

Code type	Codes for COVID-related medical encounters
**ICD-10-CM**	J1282, U071, U072, B34.2
**SNOMED**	1119302008, 119731000146105, 119741000146102, 119751000146104, 119981000146107, 1240411000000107, 1240521000000100, 1240531000000103, 1240541000000107, 1240561000000108, 1240581000000104, 674814021000119106, 840533007, 840534001, 840536004, 840539006, 866151004, 866152006, 870577009, 870588003, 870589006, 870590002, 870591003, 871562009

EHR, electronic health records; ICD, international classification of diseases; SNOMED, systematized nomenclature of medicine clinical terms.

Table S3. List of potential confounding baseline variables which were weighted using propensity weighting scoring prior to data analysis,

Variable	Variable Type	Categories
**Age**[a]	Categorical	5-year range from 25 years onwards (i.e., 18–24, 25–29, 30–34, 35–39, etc.)
**Sex**	Categorical	Female, Male
**Race**	Categorical	• Black or African American<br>• White<br>• Other<br>• Not Reported
**Ethnicity**	Categorical	• Hispanic<br>• Non-Hispanic<br>• Not Reported
**Insurance Type**	Categorical	• Commercial<br>• Medicaid<br>• Medicare Advanced<br>• Medicare FFS<br>• Other<br>• Unknown
**Geographic region**[b]	Categorical	• **Northeast** (Connecticut, Maine, Massachusetts, New Hampshire, New Jersey, New York, Pennsylvania, Rhode Island, Vermont)<br>• **Midwest** (Illinois, Indiana, Iowa, Michigan, Minnesota, Missouri, Nebraska, North Dakota, Ohio, Kansas, South Dakota, Wisconsin)<br>• **South** (Alabama, Arkansas, Delaware, District of Columbia, Florida, Georgia, Kentucky, Louisiana, Maryland, Mississippi, North Carolina, Oklahoma, South Carolina, Tennessee, Texas, Virginia, West Virginia)<br>• **West** (Alaska, Arizona, California, Colorado, Hawaii, Idaho, Montana, Nevada, New Mexico, Oregon, Utah, Washington, Wyoming)<br>• **Other**<br>• **Not Reported/missing**
**Underlying medical conditions (with increased risk for severe COVID-19 outcomes**[c] **)**	Categorical	• Asthma<br>• Cancer<br>• Cerebrovascular disease<br>• Chronic kidney disease<br>• Chronic lung diseases<br>• Chronic liver disease<br>• Cystic fibrosis<br>• Diabetes mellitus, types 1 and 2<br>• Disabilities, including Down syndrome<br>• Heart conditions<br>• HIV<br>• Mental health conditions<br>• Neurological conditions<br>• Obesity (BMI >30 kg/m^2)

		• Physical inactivity<br>• Pregnancy and recent pregnancy<br>• Primary immunodeficiencies<br>• Smoking, current and former<br>• Solid organ or blood stem cell transplantation<br>• Tuberculosis<br>• Use of corticosteroids or other immunosuppressive medications
**Number of outpatient visits in year prior to index date**	Continuous	–
**Number of all-cause hospital admission in year prior to index data**	Continuous	–
**Data source of recorded vaccination[d]**	Categorical	• Inpatient claim<br>• Outpatient claim<br>• Outpatient EHR data<br>• Pharmacy claim
**Primary series vaccination**	Categorical	• Homologous = same brand as bivalent dose<br>• Heterologous = different brand from bivalent dose<br>• Not documented
**Time since last COVID-19 monovalent vaccination**	Categorical	• Less than 3 months<br>• 3 to 6 months<br>• 6 months or more<br>• Not documented
**Time since last COVID-19 infection**	Categorical	• Less than 4 months<br>• 4 to 6 months<br>• 6 months or more<br>• Not documented
**Month of index date**	Categorical	Calendar month of index date

BMI, body mass index; CDC, Centers for Disease Control and Prevention; EHR, electronic health records; FFS, fee for service. [a]Participant age was capped at ≤89 years to prevent possible patient re-identification. [b]Categorized into one of five mutually exclusive geographic locations in the U.S. based on regional census divisions (https://www2.census.gov/geo/pdfs/maps-data/maps/reference/us_regdiv.pdf). [c]As per CDC guidelines: https://www.cdc.gov/coronavirus/2019-ncov/hcp/clinical-care/underlyingconditions.html#print. [d]Counts of patients by recorded vaccination source for each vaccine type are reported. These counts were mutually exclusive and decided in a hierarchical manner.

Table S4. Baseline characteristics (post-weighting) of individuals ≥50 years vaccinated with the mRNA-1273.222 or BNT162b2 Bivalent vaccine.

		mRNA-1273.222	BNT162b2 Bivalent	SMD
**Number of patients**		773,586	1,178,604	
**Age at index, mean (SD)**		67 (9.8)	67 (9.8)	0.0096
**Sex**	Female	441,890 (57.1)	674,578 (57.2)	0.0023
	Male	331,696 (42.9)	504,026 (42.8)	
**Race**	Black	34,192 (4.4)	53,214 (4.5)	0.0048
	Other	28,091 (3.6)	42,554 (3.6)	
	White	330,585 (42.7)	502,282 (42.6)	
	Unknown	380,720 (49.2)	580,554 (49.3)	
**Ethnicity**	Hispanic	27,889 (3.6)	43,003 (3.6)	0.0037
	Non-Hispanic	628,014 (81.2)	955,114 (81.0)	
	Unknown	117,684 (15.2)	180,488 (15.3)	
**Geographic region**	Midwest	154,244 (19.9)	246,754 (20.9)	0.0257
	Northeast	210,073 (27.2)	318,540 (27.0)	
	South	228,525 (29.5)	340,656 (28.9)	
	West	133,783 (17.3)	202,010 (17.1)	
	Unknown	46,961 (6.1)	70,645 (6.0)	
**Month of index date**	08-2022	7 (<0.1)	10 (<0.1)	0.0281
	09-2022	193,716 (25.0)	309,209 (26.2)	
	10-2022	293,896 (38.0)	443,231 (37.6)	
	11-2022	159,921 (20.7)	237,589 (20.2)	
	12-2022	88,664 (11.5)	132,321 (11.2)	
	1-2023	29,837 (3.9)	44,812 (3.8)	
	2-2023	7,545 (1.0)	11,433 (1.0)	
**Primary series COVID-19 vaccine**	Heterologous	65,270 (8.4)	108,088 (9.2)	0.0410
	Homologous	184,827 (23.9)	263,818 (22.4)	
	Not reported	523,490 (67.7)	806,699 (68.4)	
**Time since last COVID-19 monovalent vaccination**	≤90 days	11,598 (1.5)	17,290 (1.5)	0.0400
	91–180 days	159,810 (20.7)	224,925 (19.1)	
	>180 days	418,407 (54.1)	649,236 (55.1)	
	Not reported	183,772 (23.8)	287,154 (24.4)	
**Time since last COVID-19 infection**	≤120 days	26,820 (3.5)	41,276 (3.5)	0.0084
	121–180 days	14,095 (1.8)	21,416 (1.8)	
	>180 days	55,039 (7.1)	86,306 (7.3)	
	Not reported	677,633 (87.6)	10,29,606 (87.4)	
**Underlying medical conditions**	Asthma	66,720 (8.6)	102,181 (8.7)	0.0016
	Cancer	276,033 (35.7)	418,297 (35.5)	0.0040
	Cerebrovascular disease	54,803 (7.1)	84,117 (7.1)	0.0021
	Chronic lung disease	77,622 (10.0)	118,678 (10.1)	0.0012
	Chronic liver disease	10,348 (1.3)	15,948 (1.4)	0.0013

	CKD	85,312 (11)	130,593 (11.1)	0.0017
	Cystic fibrosis	100 (<0.1)	141 (<0.1)	0.0008
	Diabetes type 1 or 2	189,989 (24.6)	290,136 (24.6)	0.0013
	Disability	38,177 (4.9)	58,560 (5.0)	0.0015
	Heart conditions	137,395 (17.8)	209,653 (17.8)	0.0007
	HIV	4,145 (0.5)	6,349 (0.5)	0.0004
	Mental health disorders	116,509 (15.1)	181,524 (15.4)	0.0095
	Neurological conditions	22,418 (2.9)	36,092 (3.1)	0.0097
	Obesity	167,583 (21.7)	257,071 (21.8)	0.0036
	Primary immunodeficiencies	57,071 (7.4)	86,869 (7.4)	0.0003
	Pregnancy[a]	15 (<0.1)	23 (<0.1)	0.0001
	Physical inactivity	786 (0.1)	1,215 (0.1)	0.0005
	Smoking[b]	109,796 (14.2)	168,895 (14.3)	0.0039
	Solid organ or hematopoietic stem cell transplant	8,011 (1.0)	12,374 (1.0)	0.0014
	Tuberculosis	259 (<0.1)	395 (<0.1)	0.000
	Use of immunosuppressants	45,087 (5.8)	68,657 (5.8)	0.0001

Data are presented as n (%) unless otherwise stated. CKD, chronic kidney disease; SD, standard deviation; SMD, standardized mean difference. [a]Includes recent pregnancy. [b]Includes current and former smokers.

Table S5. Baseline characteristics (post-weighting) of individuals ≥65 years vaccinated with the mRNA-1273.222 or BNT162b2 Bivalent vaccine.

		mRNA-1273.222	BNT162b2 Bivalent	SMD
**Number of patients**		445,994	641,195	
**Age at index, mean (SD)**		74 (6.6)	74 (6.7)	0.0023
**Sex**	Female	253,468 (56.8)	365,297 (57.0)	0.0028
	Male	192,525 (43.2)	275,898 (43.0)	
**Race**	Black	19,132 (4.3)	27,806 (4.3)	0.0030
	Other	13,813 (3.1)	19,675 (3.1)	
	White	204,836 (45.9)	294,383 (45.9)	
	Unknown	208,213 (46.7)	299,331 (46.7)	
**Ethnicity**	Hispanic	14,184 (3.2)	20,630 (3.2)	0.0031
	Non-Hispanic	365,189 (81.9)	524,332 (81.8)	
	Unknown	66,621 (14.9)	96,233 (15.0)	
**Geographic region**	Midwest	88,084 (19.8)	132,347 (20.6)	0.0231
	Northeast	133,004 (29.8)	190,573 (29.7)	
	South	130,925 (29.4)	184,547 (28.8)	
	West	67,466 (15.1)	95,983 (15.0)	
	Unknown	26,514 (5.9)	37,745 (5.9)	
**Month of index date**	08-2022	4 (<0.1)	5 (<0.1)	0.0217
	09-2022	121,214 (27.2)	180,378 (28.1)	
	10-2022	170,795 (38.3)	243,450 (38)	
	11-2022	85,888 (19.3)	120,926 (18.9)	
	12-2022	46,044 (10.3)	65,199 (10.2)	
	1-2023	17650 (4.0)	25,039 (3.9)	
	2-2023	4,399 (1.0)	6,198 (1.0)	
**Primary series COVID-19 vaccine**	Heterologous	23,370 (5.2)	43,556 (6.8)	0.0764
	Homologous	88,844 (19.9)	115,802 (18.1)	
	Not reported	333,781 (74.8)	481,837 (75.1)	
**Time since last COVID-19 monovalent vaccination**	≤90 days	6,827 (1.5)	9,565 (1.5)	0.0418
	91–180 days	102,382 (23.0)	136,674 (21.3)	
	>180 days	203,504 (45.6)	302,506 (47.2)	
	Not reported	133,281 (29.9)	192,450 (30.0)	
**Time since last COVID-19 infection**	≤120 days	15,150 (3.4)	22,238 (3.5)	0.0103
	121–180 days	7,257 (1.6)	10,493 (1.6)	
	>180 days	28,855 (6.5)	42,960 (6.7)	
	Not reported	394,732 (88.5)	565,504 (88.2)	
**Underlying medical conditions**	Asthma	36,304 (8.1)	52,433 (8.2)	0.0014
	Cancer	173,386 (38.9)	248,505 (38.8)	0.0025

	Cerebrovascular disease	44,071 (9.9)	64,071 (10.0)	0.0037
	Chronic lung disease	59,176 (13.3)	86,016 (13.4)	0.0043
	Chronic liver disease	5,852 (1.3)	8,542 (1.3)	0.0018
	CKD	71,141 (16)	103,363 (16.1)	0.0046
	Cystic fibrosis	48 (<0.1)	67 (<0.1)	0.0003
	Diabetes type 1 or 2	125,880 (28.2)	181,558 (28.3)	0.0020
	Disability	19,111 (4.3)	27,624 (4.3)	0.0011
	Heart conditions	107,398 (24.1)	155,468 (24.2)	0.0039
	HIV	1,217 (0.3)	1,766 (0.3)	0.0005
	Mental health disorders	64,813 (14.5)	95,743 (14.9)	0.0113
	Neurological conditions	21,113 (4.7)	32,188 (5)	0.0133
	Obesity	92,563 (20.8)	133,914 (20.9)	0.0032
	Primary immunodeficiencies	33,885 (7.6)	48,689 (7.6)	0.0002
	Physical inactivity	498 (0.1)	734 (0.1)	0.0009
	Smoking[a]	68,883 (15.4)	100,317 (15.6)	0.0055
	Solid organ or hematopoietic stem cell transplant	5,561 (1.2)	8,054 (1.3)	0.0008
	Tuberculosis	167 (<0.1)	237 (<0.1)	0.0003
	Use of immunosuppressants	27,115 (6.1)	38,984 (6.1)	0.0000

Data are presented as n (%) unless otherwise stated. CKD, chronic kidney disease; SD, standard deviation; SMD, standardized mean difference. [a]Includes current and former smokers.

Table S6. Baseline characteristics (post-weighting) of individuals hospitalized for COVID-19 vaccinated with the mRNA-1273.222 or BNT162b2 Bivalent vaccine.

		mRNA-1273.222	BNT162b2 Bivalent	SMD
**Number of patients**		1,032	1,855	
**Age at index, mean (SD)**		71 (14.0)	72 (14.2)	0.0837
**Sex**	Female	524 (50.8)	960 (51.8)	0.0192
	Male	508 (49.2)	895 (48.2)	
**Race**	Black	51 (5.0)	84 (4.5)	0.1134
	Other	33 (3.2)	42 (2.3)	
	White	426 (41.3)	693 (37.4)	
	Unknown	522 (50.5)	1036 (55.9)	
**Ethnicity**	Hispanic	42 (4.1)	60 (3.2)	0.0452
	Non-Hispanic	872 (84.5)	1586 (85.5)	
	Unknown	119 (11.5)	210 (11.3)	
**Geographic region**	Midwest	207 (20.1)	418 (22.5)	0.089
	Northeast	327 (31.7)	589 (31.8)	
	South	276 (26.8)	435 (23.5)	
	West	139 (13.5)	251 (13.5)	
	Unknown	83 (8.0)	162 (8.7)	
**Month of index date**	08-2022	0 (0.0)	0 (0.0)	0.0965
	09-2022	334 (32.4)	665 (35.9)	
	10-2022	452 (43.8)	788 (42.5)	
	11-2022	190 (18.4)	290 (15.7)	
	12-2022	47 (4.6)	97 (5.2)	
	1-2023	9 (0.8)	14 (0.8)	
	2-2023	0 (0.0)	0 (0.0)	
**Primary series COVID-19 vaccine**	Heterologous	75 (7.3)	172 (9.3)	0.0719
	Homologous	201 (19.4)	354 (19.1)	
	Not reported	756 (73.3)	1329 (71.7)	
**Time since last COVID-19 monovalent vaccination**	≤90 days	19 (1.8)	45 (2.4)	0.0571
	91–180 days	224 (21.7)	401 (21.6)	
	>180 days	506 (49.0)	872 (47.0)	
	Not reported	283 (27.4)	537 (29.0)	
**Time since last COVID-19 infection**	≤120 days	105 (10.2)	219 (11.8)	0.0643
	121–180 days	20 (1.9)	29 (1.6)	
	>180 days	100 (9.7)	193 (10.4)	
	Not reported	807 (78.2)	1414 (76.2)	
**Underlying medical conditions**	Asthma	173 (16.8)	277 (14.9)	0.0497
	Cancer	437 (42.3)	828 (44.6)	0.0457
	Cerebrovascular disease	204 (19.8)	390 (21.0)	0.0309
	Chronic lung disease	326 (31.5)	655 (35.3)	0.0796

	Chronic liver disease	39 (3.8)	74 (4.0)	0.0094
	CKD	343 (33.2)	640 (34.5)	0.0265
	Diabetes type 1 or 2	417 (40.4)	817 (44.0)	0.0748
	Disability	106 (10.3)	182 (9.8)	0.0153
	Heart conditions	481 (46.6)	909 (49.0)	0.0477
	HIV	9 (0.9)	12 (0.6)	0.0242
	Mental health disorders	287 (27.8)	554 (29.8)	0.0445
	Neurological conditions	137 (13.2)	274 (14.8)	0.0448
	Obesity	304 (29.5)	571 (30.8)	0.0278
	Primary immunodeficiencies	155 (15.1)	279 (15.0)	0.0002
	Pregnancy[a]	30 (2.9)	38 (2.1)	0.0559
	Physical inactivity	4 (0.4)	2 (<0.1)	0.0611
	Smoking[b]	303 (29.4)	568 (30.6)	0.0263
	Solid organ or hematopoietic stem cell transplant	67 (6.5)	139 (7.5)	0.0380
	Tuberculosis	1 (0.1)	4 (0.2)	0.0147
	Use of immunosuppressants	118 (11.4)	200 (10.8)	0.0191

Data are presented as n (%) unless otherwise stated. [a]Includes recent pregnancy. [b]Includes current and former smokers. CKD, chronic kidney disease; SD, standard deviation; SMD, standardized mean difference.

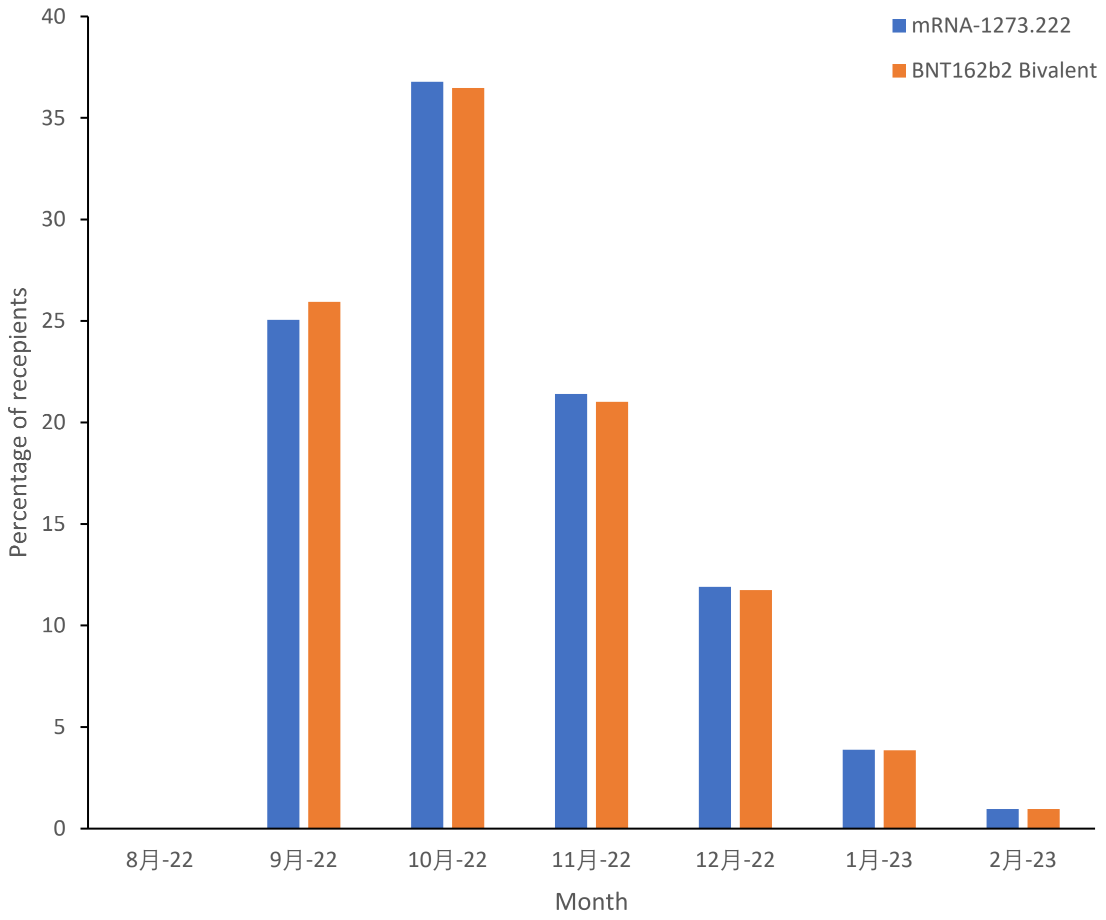

Figure S1. Percentage of individuals included in the weighted analysis by month of vaccination.

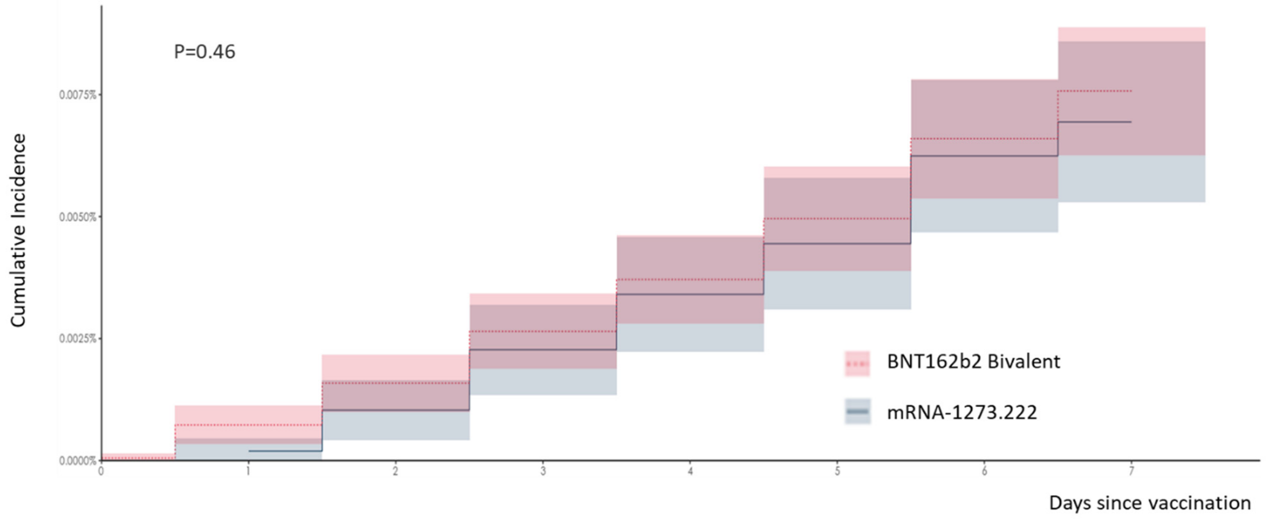

Figure S2. Negative control Kaplan–Meier curves for COVID-19–related hospitalization rates 7 days prior to CED in recipients of the mRNA-1273.222 or BNT162b2 Bivalent vaccine. The dashed pink line represents the risk curve for BNT162b2 Bivalent. The solid blue line represents the risk curve for mRNA-1273-222. Shaded areas represent 95% confidence intervals.

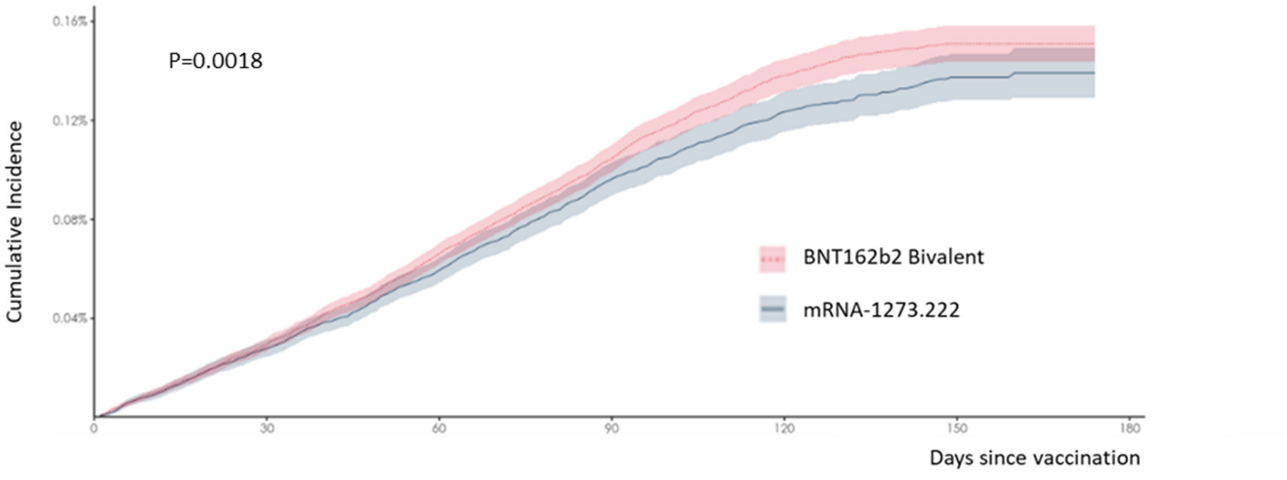

Number at risk

	0d	30d	60d	90d	120d	150d
mRNA-1273.222 bivalent	1,034,538	967,332	850,932	662,711	414,249	140,204
BNT162b2 bivalent	1,670,666	1,565,594	1,380,493	1,075,062	683,810	233,213

Cumulative number of events

	0d	30d	60d	90d	120d	150d
mRNA-1273.222 bivalent	0	280	568	845	993	1,031
BNT162b2 bivalent	0	475	1,019	1,487	1,790	1,855

Figure S3. Kaplan–Meier curves of COVID-19–related hospitalizations over time in recipients of the mRNA-1273.222 or BNT162b2 Bivalent vaccine.

Supplementary References

1. Nguyen VH, Boileau C, Bogdanov A, et al. Relative effectiveness of BNT162b2, mRNA-1273, and Ad26.COV2.S vaccines and homologous boosting in preventing COVID-19 in adults in the US. Open Forum Infectious Diseases 2023:ofad288.

2. Boikos C, McGovern I, Ortiz JR, Puig-Barberà J, Versage E, Haag M. Relative Vaccine Effectiveness of Adjuvanted Trivalent Influenza Vaccine over Three Consecutive Influenza Seasons in the United States. Vaccines (Basel) 2022;10.

3. Imran M, Ortiz JR, McLean HQ, et al. Relative Effectiveness of Cell-based Versus Egg-based Quadrivalent Influenza Vaccines in Children and Adolescents in the United States During the 2019-2020 Influenza Season. Pediatr Infect Dis J 2022;41:769-74.

4. Holm S. A Simple Sequentially Rejective Multiple Test Procedure. Scandinavian Journal of Statistics 1979;6:65-70.
